# Body Fat Percentages by Dual-energy X-ray Absorptiometry Corresponding to Body Mass Index Cutoffs for Overweight and Obesity in Indian Children

**DOI:** 10.4137/cmped.s3446

**Published:** 2009-11-30

**Authors:** Deepa Pandit, Shashi Chiplonkar, Anuradha Khadilkar, Vaman Khadilkar, Veena Ekbote

**Affiliations:** 1 Agharkar Research Institute, Agarakar Road, Pune, India.; 2 HC Jehangir Medical Research Institute, Sasson Road, Pune, India. Email: drsjc_1@yahoo.com

**Keywords:** BMI, percent body fat, overweight, children, lunar DXA

## Abstract

**Background:**

Indians are suspected to have higher body fat percent at a given body mass index (BMI) than their western counterparts.

**Objective:**

To estimate percent body fat in apparently healthy Indian children and adolescents by dual-energy X-ray absorptiometry (DXA) and explore linkages of BMI with body fat percent for better health risk assessment.

**Methods:**

Age, weight, height of 316 boys and 250 girls (6–17 years) were recorded. Body composition was measured by dual-energy X-ray absorptiometry (DXA). High adiposity was defined as body fat percent (BF%) > McCarthy’s 85th percentile of body fat reference data. Receiver operating characteristic analysis (ROC) was carried out for CDC BMI Z score for it’s ability to judge excess fatness.

**Results:**

High BF% was seen in 38.5% boys and 54.0% girls (p < 0.05). Percentage of obese children as defined by the BMI cutoffs of International Obesity Task Force (IOTF) (2.1% for boys and 6.9% for girls) was lower than that using Indian (13.7% for boys and 20.9% for girls) and CDC (14.1% for boys and 20.9% for girls) cutoffs. The point closest to one on the ROC curves of CDC BMI Z-scores indicated high adiposity at BMI cutoff of 22 at the age of 17 yr in both the genders.

**Conclusions:**

Higher body fat percentage is associated with lower BMI values in Indian children.

## Introduction

Childhood obesity has become a major public health concern over the past decade.^1^ In India, there is an increasing trend to overweightness and obesity in the growing age due to sedentary lifestyle and changed food habits especially in urban affluent society.^2^,^3^ The body mass index (BMI) is used widely as an indicator of the risk of overweight or obesity, because of the relative ease and accuracy of the basic measurement. For evaluation of thinness as well as obesity the WHO expert group has also recommended body ponderosity indices particularly BMI.^4^ International Obesity task Force (IOTF) published such an analysis of an international database of children aged 2–18 y from 6 countries and BMI cutoffs were provided for every half-year of age in both sexes.^5^ Additionally there are new growth charts from the US Centers for Disease Control and Prevention (CDC), which include age- and sex-specific BMI reference values for children and adolescents aged 2–20 y.^6^ However, in clinical practice, the important question is whether the criteria for overweight and obesity that are based only on the upper portion of the BMI distribution (i.e. 85th or 95th percentile) identify correctly the fattest children and those at greatest health risk.^7^ An important limitation of the BMI as a measure of obesity is that it tends to ignore the distinction between fat and fat-free mass.

The ability of BMI to correctly classify children as obese in relation to different measures of body composition has been investigated by many studies.^8^–^10^ These studies have generally reported that while BMI has reasonable efficacy for identifying children who are truly lean, it appears to be relatively insensitive at identifying children who are truly fat. Although BMI is strongly correlated with adiposity in children, it may not be suitable for use because the tracing of BMI from childhood to adulthood reflects continuities in “body build” rather than adiposity.^11^ Further research is therefore necessary to examine the typical levels of adiposity associated with BMI cutoffs of overweight and obesity in childhood and ascertain whether these cutoffs can discriminate excess body fatness using measurements of percentage body fat (%BF).

Due to ethnic differences Asians are shown to have higher body fat than the Caucasians.^12^ Various studies have suggested that adult Asian populations have different associations between BMI, percentage of body fat, and health risks than do European populations.^13^ It has also been suggested that a specific BMI reflects a higher percentage of body fat in Asians than in European populations. Many studies have increasingly emphasized the importance of body fat in determining cardiovascular risk status.^14^,^15^ Studies relating BMI with body fat measurement by dual-energy X-ray absorptiometry (DXA) are few and are mostly done on Western population.^15^–^17^ Indians are suspected to have higher body fat percentage than their western counterparts.^18^ Available data indicates that body fat percentage in Indian children and adolescents is high as measured by bio-electrical impedance analyser (BIA) than their Western counterparts.^3^,^19^ However DXA reports on Indian children are scarce. Our aim was therefore to estimate percent body fat in apparently healthy Indian children and adolescents by DXA and explore linkages of BMI with body fat percent in Indian children and adolescents for better health risk assessment.

## Methods

A cross sectional health survey was conducted in private and public schools, private clinics in and around Pune city and health check up at Jehangir hospital. In total, 316 boys and 250 girls (6–17 years) participated in the study on a voluntary basis. An informed written consent from their parents/guardians was obtained. The research protocol was approved by the Ethics Committee of Hirabai Cowasji Jehangir Medical Research Institute (HCJMRI), Pune, India.

### Measurements

Weight and height were measured by a trained investigator in the morning with participants in light indoor clothes without shoes. Height measurement was done with a stadiometer to the nearest 1 mm. Weight was measured on electronic digital scale to the nearest 0.1 kg. Body mass index was calculated as Weight (kg) divided by Height squared (m^2^).

Dual Energy X-ray Absorptiometry (DXA) allows the separation of body mass into fat mass (FM), lean tissue mass (LTM) and bone mineral content (BMC). DXA measurements were performed using Lunar DPX-PRO total body pencil beam Densitometer (GE Healthcare, Wisconsin, USA) using a medium mode scan (software encore 2005 version 9.30.044). The precision of repeat measurements in adults with DPX Pro is reported to be 2.0% for Fat Mass.^20^ We have standardized our measurements in adolescents by running daily quality assurance scans. The manufacturer’s appointed service engineer was requested to review the calibration data and provide a scanner maintenance check to ensure the system’s performance before the first subject was scanned, and to confirm that no instrumentation drift occurred. All scans and scan analysis were performed by the same operator. The precision of repeat measurements of body fat percent in adolescents was observed to be 1.1% in the present study.

### Statistical methods

Analyses were performed for boys and girls separately using SPSS software for Windows (version 11.0, 2001, SPSS Inc, Chicago, IL). Considering their pubertal growth years, the children were classified in four age groups, (6–8, 9–11, 12–14, 15–17) and gender differences in body measurements were tested using one-way ANOVA with post hoc Tukey’s test. BMI-based classification for obesity by IOTF, CDC and Agrawal (AGR) were used to assess overweight or obesity in children and adolescents (Table 1). Concordance between different BMI cutoffs with respect to prevalence of overweight and obesity was calculated using Kappa coefficient. Gender differences between prevalence of overweight and obesity were tested using chi square test.

High adiposity was defined as levels of BF% greater than the 85th percentile of McCarthy’s BF% reference data for each yearly age-sex group.^21^ Receiver operating characteristic (ROC) analysis was carried out to evaluate the general performance of BMI in reflecting body fatness. ROC curve is a plot of the true positive rate (sensitivity) against the false positive rate (1-specificity) across range of values from the diagnostic test. The decision threshold is the criterion value with the highest accuracy that maximizes the sum of the sensitivity and specificity.^22^,^23^ In the present study, the diagnostic test is the excess adiposity according to McCarthy’s 85th percentile of BF% and sensitivity of CDC BMI Z score for adiposity was examined. The areas under each ROC curve (AUC) and their 95% confidence intervals (CI) were estimated to compare the relative ability of BMI to estimate high percentage fat mass. The lower area under the curve explains less sensitive and specific optimal cutoffs. Positive predictive value was computed as a probability that the excess fatness is present when the BMI is high and negative predictive value was calculated as a probability that the excess fatness is not present when the BMI is normal. BMI Z score was calculated using L (Lambda), M (mu) and S (sigma) values of CDC BMI charts for each age-sex group. ROC analysis was carried out to obtain a threshold of BMI Z score to correctly estimate risk of high adiposity.

## Results

Mean BMI and BF% of boys and girls for each yearly age group are given in Table 2. Mean BMI and body fat% was higher in adolescents than in young children in both boys and girls. Mean BMI of girls was significantly higher than boys after 12 years of age (p < 0.05). However for boys, BF% was lower after the age of 13 yr and remained same till 17 yr. According to McCarthy’s cutoffs for body fat in childhood 38.5% boys and 54.0% girls had high percentage body fat values (p < 0.05). Overall correlation coefficient between BMI and BF% was statistically significant in boys (r = 0.89) and in girls (r = 0.89) after adjusting for age (p < 0.01).

Percent prevalence of overweight and obesity was computed using cutoffs of 3 reference data sets (Table 1). Prevalence of obesity using CDC and AGR cutoffs was similar and higher than IOTF (Table 3). Prevalence of overweight (9.6%) and obesity (14.1%) using Indian BMI cutoffs was lower in boys than that in overweight (13.9%) and obese (18.8%) girls. Prevalence of obesity with IOTF cutoffs (2.1% for boys and 6.9% for girls) was lower than that of AGR (13.7% for boys and 20.9% for girls) and CDC (14.1% for boys and 20.9% for girls) cutoffs. Concordance between IOTF and CDC (kappa = 0.31) and IOTF and AGR (kappa = 0.27) was low (p < 0.01) and that between CDC and AGR was high (kappa = 0.88, p < 0.01). This indicates the probability of misclassification by IOTF to be high than by using CDC and AGR cutoffs.

**Figure 1:**
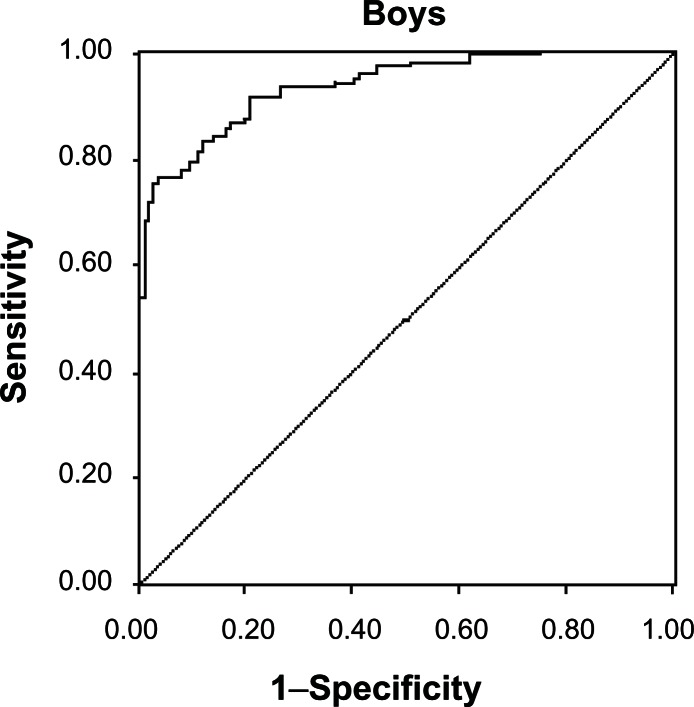
ROC curve of CDC BMI Z score for boys with McCarthy’s body fat cutoffs. Sensitivity is 0.77, Specificity is 0.97, Area under the curve is 0.919 (0.882–0.956), Positive and negative predictive values are 95.02 and 80.8.

**Figure 2:**
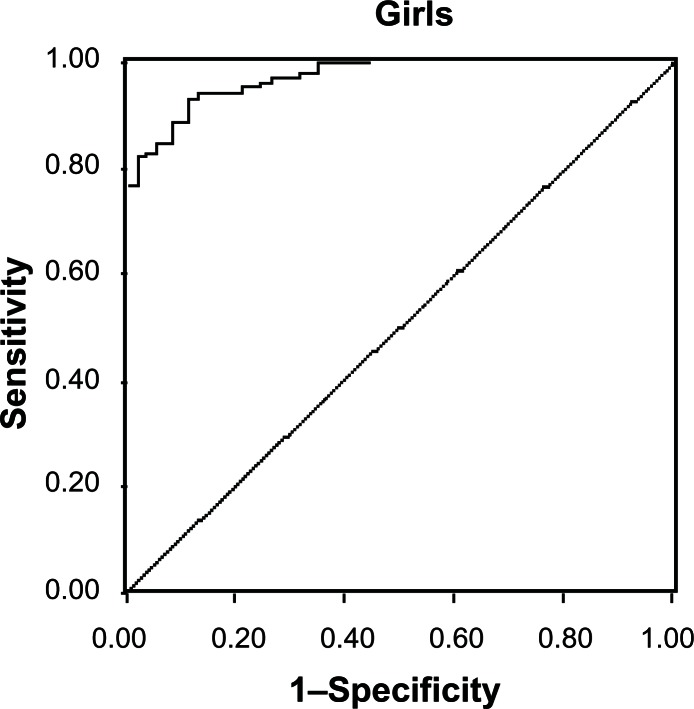
ROC curve of CDC BMI Zscore for girls with McCarthy’s body fat cutoffs. Sensitivity is 0.83, Specificity is 0.97, Area under the curve is 0.961 (0.939–0.983), Positive and negative predictive values are 96.5 and 85.2.

ROC analysis of CDC BMI Z scores showed a higher sensitivity (77% and 83% for boys and girls respectively) and specificity (97% for boys and girls) using McCarthy’s cutoffs for both the genders. Positive predictive values for boys and girls indicated that 95% of the children, who have high adiposity, may be identified correctly as overweight or obese when BMI Z score is used (Figs. 1 and 2). The point closest to one on the ROC curves (Figs. 1 and 2) is represented by BMI Z-scores of 0.11 in boys and 0.07 in girls using McCarthy’s cutoffs. One hundred boys (31.6%) had BMI Z score values greater than 0.11 and 112 girls (44.6%) had BMI values greater than 0.07. This corresponds to a BMI cutoff of 22 for overweight for boys and girls at the age of 17 yr. At the age of 17 yr BMI cutoff of overweight in boys is around 24 in AGR, CDC and IOTF and in girls 23 for AGR and 25 for CDC and IOTF (Table 1).

## Discussion

Our study findings suggest that according to McCarthy’s cutoff overall percentage of high adiposity was 38.5% in boys and 54% in girls. At the age of 17 years, the 85th percentile of body fat% in our data was found to be 28.8% in boys and 42.5% in girls. These are higher than the McCarthy’s values of 85th percentile of body fat% at the age of 17 yr, i.e. 20.1% for boys and 30.4% for girls. When assessed against BMI cutoff at the age of 17 yr in both the sexes, for a BMI of 22, the corresponding body fat% was of the order of 24.1 and 34.3% in boys and girls respectively.

Our data provides first hand estimates of body fat percent by DXA in Indian children and adolescents. Percent body fat by DXA in Western children (7 to 18 yr.) were 17% to 19% in boys and 22% to 31% in girls.^15^,^16^,^24^ These are lower than the mean BF% of 16.1% to 24.8% in boys and 27.7% to 35.4% in girls of similar age range in the present study. Percent body fat values of girls and boys in our study are similar to those reported by other studies on Indian adolescents using BIA^19^ while in case of girls BF% is higher in our data than that of affluent class girls of same age range.^3^ Thus our data supports the finding that Indian children and adolescents are having higher body fat% than their Western counterparts. Further correlation of percent body fat and BMI after adjusting for age in the present study was of the similar order that has been reported by other studies.^7^,^19^

ROC analysis of BMI Z scores was used to estimate the exact BMI cutoffs for excess fatness, which gave 22 as BMI cutoff in both boys and girls at the age of 17 years. This showed a high sensitivity (0.77 for boys and 0.83 for girls) and specificity (0.97). Similar values of sensitivity and specificity of BMI Z score with body fat% were reported by Taylor, 2003^24^ on adolescents from New Zealand. Though BMI Z score is a good indicator for assessing excess fatness, computing Z scores using Agarwal’s data is not feasible as it does not provide LMS values. With the existing BMI cutoff of 25, associated body fat% in our data is 36% in boys and 43.8% in girls. ROC analysis of BMI Z score using >85th percentile of percent body fat by McCarthy as gold standard revealed that the cutoffs for BMI need to be lower by at least 3 points than the existing adult equivalent cutoffs of 25 for overweight category.

Many studies on children and adolescents have reported low sensitivity and high specificity of conventional BMI cut-off point in detecting overweight.^10^,^19^,^25^ However these studies have estimated body fat using skinfold thickness or BIA. In the present study, we have examined sensitivity and specificity of BMI Z score using DXA estimates of percent body fat. The sensitivities of the 85th and 95th BMI percentiles on the CDC 2000 growth charts in identifying correctly the fat children range from 54% to 100% in several studies, and the corresponding specificities range from 67% to 99%.^17^,^26^ Our values of sensitivity and specificity for CDC BMI Z scores agree well with these studies. Our findings suggest that higher body fat percent is associated with lower BMI values in Indian children and adolescents.

Ellis et al have reported that the DXA overestimates BF% at higher values and underestimates at lower values when compared with the four-compartment model (4-CM) as the “gold standard”.^27^ Although this multicompartment approach is preferred, it is impractical for most laboratories because of the cost and necessary equipment needed.^28^ Because of the predictable relationship between DXA and 4-CM for %BF measurement and its ease of use, it has been proposed that DXA has the capacity for clinical application including prediction of metabolic abnormalities associated with excess%BF in pediatrics.^29^ Using the regression equation of Sopher 2004, which is; %BF (4-CM) = 0.7739* %BF DXA (by Lunar)+ 4.128, BF% by DXA in our data was 26.4% in boys and 37.0% in girls. This is still higher than the McCarthy’s cut offs at the age of 17 yr. We have compared our results on Lunar with other studies on Lunar machines for DXA.

Our results are supported by the view that BMI criteria by themselves are insufficient to identify children who are most likely to have clusters of risk factors and thus additional screening and assessment criteria should be applied to estimate risks.^30^ Waist circumference and skin fold thickness have been used to assess health risk,^7^ however no overall differences were found in the ability of BMI, waist circumference, and triceps/subscapular skinfold ratio cutoff points to identify correctly Spanish children with the metabolic syndrome.^31^

A limitation of our study is relatively small sample size in each age-sex group and our data pertains to a section of urban population of western India. However results of the present study can be used as the base line information for assessing usefulness of body fat-linked BMI cutoffs in predicting increased metabolic risk in children and adolescents.

## Figures and Tables

**Table 1:** Age-sex specific cutoffs for overweight and obesity based on BMI by different classifications.

**Age (yr)**	**Agarwal**		**CDC**		**IOTF**	
	**BMI percentile**		**BMI percentile**		**BMI**	
	**85th**	**95th**	**85th**	**95th**	**25**	**30**
**Boys**						
6	15.9	17.8	17.0	18.4	17.5	19.7
7	16.4	18.8	17.4	19.1	17.9	20.6
8	17.0	19.7	17.9	20.0	18.4	21.6
9	17.3	21.0	18.6	21.0	19.1	22.7
10	18.5	22.1	19.3	22.1	19.8	24.0
11	19.1	23.4	20.1	23.2	20.5	25.1
12	19.8	23.8	21.0	24.2	21.2	26.0
13	20.4	25.3	21.8	25.1	21.9	26.8
14	21.1	25.3	22.6	26.0	22.6	27.6
15	22.0	27.3	23.4	26.8	23.2	28.3
16	22.7	27.6	24.2	27.5	23.9	28.8
17	24.4	26.8	24.9	28.2	24.4	29.4
**Girls**						
6	16.0	18.8	17.0	18.8	17.3	19.6
7	16.6	19.7	17.6	19.6	17.7	20.5
8	18.0	21.4	18.3	20.6	18.3	21.5
9	18.0	21.7	19.1	21.8	19.0	22.8
10	19.9	23.2	19.9	22.9	19.8	24.1
11	20.6	24.5	20.8	24.1	20.7	25.4
12	21.9	25.7	21.7	25.2	21.6	26.6
13	22.6	27.1	22.5	26.2	22.5	27.7
14	23.0	27.4	23.3	27.2	23.3	28.5
15	23.6	27.7	24.0	28.1	23.9	29.1
16	23.7	27.4	24.6	28.9	24.3	29.4
17	23.0	25.9	25.2	29.6	24.7	29.6

**Source:** Agarwal KN, Saxena A, Bansal AK, Agarwal DK. Physical Growth Assessment in Adolescence. *Ind Pediatr*. 2001;38:1217–35. CDC BMI charts, developed by the National Center for Health Statistics in collaboration with the National Center for Chronic Disease Prevention and Health Promotion 2000. http://www.cdc.gov/growthcharts. Cole TJ, Bellizzi MC, Flegal KM, Dietz WH. Establishing a standard definition for child overweight and obesity worldwide: international study. *Brit Med J*. 2000;320:1240–3.

**Table 2:** Body mass index and body fat percentage in boys and girls across different age groups.

**Boys (316)**				**Girls (250)**		
**Age groups**	**Number**	**BMI Mean ± SE**	**BF% Mean ± SE**	**Number**	**BMI Mean ± SE**	**BF% Mean ± SE**
6–7	12	13.7 ± 0.2	11.7 ± 0.8	12	17.7 ± 1.3	29.9 ± 3.5
7–8	11	16.1 ± 1.7	20.3 ± 4.5	13	15.1 ± 0.8	23.7 ± 2.8
8–9	23	16.4 ± 0.6	21.2 ± 1.9	33	16.8 ± 0.7	26.7 ± 2.2
9–10	27	17.8 ± 1.5	26.2 ± 3.6	35	16.6 ± 0.7	27.3 ± 2.0
10–11	36	16.9 ± 0.8	23.5 ± 2.2	38	18.1 ± 0.9	30.6 ± 2.3
11–12	43	17.8 ± 0.7	25.8 ± 2.3	35	19.1 ± 1.1	32.8 ± 2.4
12–13	36	19.0 ± 0.7	29.6 ± 2.3	15	22.0 ± 1.5	36.6 ± 2.7
13–14	50	18.0 ± 0.6	23.0 ± 1.9	23	22.6 ± 1.4	36.6 ± 2.6
14–15	33	18.7 ± 0.8	20.6 ± 2.3	21	24.3 ± 2.4	39.6 ± 3.4
15–16	27	19.4 ± 0.8	20.1 ± 2.3	13	21.7 ± 1.7	35.4 ± 3.1
16–17	18	19.2 ± 2.6	20.5 ± 1.4	12	22.5 ± 0.9	36.5 ± 2.0

**Table 3:** Prevalence of obesity with different classification systems and mean body fat% in each category.

	**Boys**			**Girls**		
	**Normal**	**Overweight**	**Obese**	**Normal**	**Overweight**	**Obese**
% children by IOTF BMI cutoff	89.7	8.1	2.1	82.4	10.8	6.9
BF% (Mean ± SD)	22.2 ± 11.5	45.4 ± 4.2	50.0 ± 4.7	30.5 ± 10.4	47.1 ± 3.2	51.7 ± 3.3
% children by CDC BMI cutoff	78.4	7.9	13.7	68.3	10.9	20.9
BF% (Mean ± SD)	16.4 ± 7.1	39.2 ± 8.6	45.1 ± 4.7	25.4 ± 9.3	41.8 ± 4.9	47.6 ± 4.8
% children by AGR BMI cutoff	76.2	9.6	14.1	67.3	13.9	18.8
BF% (Mean ± SD)	15.8 ± 6.4	36.4 ± 8.3	45.2 ± 4.5	25.0 ± 9.3	40.0 ± 5.7	47.8 ± 4.5

**Note:** Concordance between IOTF and CDC or IOTF and AGR was low (kappa = 0.31 and 0.27, p < 0.01) respectively and that between CDC and AGR was high (kappa = 0.88, p < 0.01).
